# Fracture and damage localization in volcanic edifice rocks from El Hierro, Stromboli and Tenerife

**DOI:** 10.1038/s41598-018-20442-w

**Published:** 2018-01-31

**Authors:** Claire E. Harnett, Philip M. Benson, Pete Rowley, Marco Fazio

**Affiliations:** 1 Institute of Geophysics and Tectonics, School of Earth and Environment, University of Leeds, Leeds, LS2 9JT UK; 2 Rock Mechanics Laboratory, School of Earth and Environmental Sciences, University of Portsmouth, Portsmouth, PO1 3QL UK; 3 School of Environmental Sciences, University of Hull, Hull, HU6 7RX UK

## Abstract

We present elastic wave velocity and strength data from a suite of three volcanic rocks taken from the volcanic edifices of El Hierro and Tenerife (Canary Islands, Spain), and Stromboli (Aeolian Islands, Italy). These rocks span a range of porosity and are taken from volcanoes that suffer from edifice instability. We measure elastic wave velocities at known incident angles to the generated through-going fault as a function of imposed strain, and examine the effect of the damage zone on P-wave velocity. Such data are important as field measurements of elastic wave tomography are key tools for understanding volcanic regions, yet hidden fractures are likely to have a significant effect on elastic wave velocity. We then use elastic wave velocity evolution to calculate concomitant crack density evolution which ranges from 0 to 0.17: highest values were correlated to the damage zone in rocks with the highest initial porosity.

## Introduction

Damage localization in brittle media is well known; the concept that even the largest faults start as the nucleation of very small defects and cracks goes back to the pioneering work of Griffith in the 1920s^1–3^. These pervasive fracture and crack networks are well known to disproportionally influence the physical properties of rocks, including permeability and elastic wave velocity^4^. In addition, any alignment of the microcrack and fracture network will add to the anisotropy measured using these methods, which has significant implications for the use of velocity surveying and other methods, which rely on some form of *a priori* velocity model^5^. Although a pervasive crack network features in many tectonic environments, the generation of a fresh network is of particular relevance in areas subject to dynamic stresses and fluids such as a volcanic edifices. A better understanding of how crack/fault fabrics influence elastic wave and other rock physic properties is becoming increasingly important given the increasing number of active surveying techniques that are being deployed to generate tomographic images of the sub-volcanic pile. Examples of such work includes attenuation of the sub-Naples area^6^, Montserrat^7^, and Mt Etna^8^. Whilst laboratory data has been collected and used for improving these types of methods, to date they have concentrated on the velocity and velocity anisotropy at fixed orientations across samples of interest^9^, and/or with respect to simple deformation experiments^10^.

Here we report a new study whereby a suite of rock samples were deformed in a triaxial deformation cell while undergoing P-wave elastic velocity surveys at 1 minute intervals. This generates a pseudo-tomography of the sample, showing not only the basic physical properties as the samples are loaded, but also the evolution of the forming fault plane. Rather than concentrate on the passive microseismic data, known as acoustic emission (AE) in a laboratory context^10^, we have focused on investigating small changes in the P-wave velocity data during the course of the experiment. The raypath angle is correlated to the eventual orientation of the generated fault plane. Such knowledge is important, as in field settings any subsurface faults and/or fabrics may not be immediately obvious, even though the survey data will be collected regardless. For dynamic geological areas such as volcanoes, which feature a diverse array of influences on rock strength and elastic wave velocity, such laboratory data would usefully inform these field-based surveys in terms of hidden damage. In this paper we apply our laboratory approach to three specific volcanic settings, which all share a common history of catastrophic flank collapse: Stromboli volcano (Aeolian Islands, Italy), and the volcanoes of Tenerife and El Hierro (Canary Islands, Spain).

Stromboli Volcano, located in the Aeolian Archipelago north of Sicily, is a continuously active composite volcano and structural surveys reveal a total of eight lateral collapses at Stromboli in the last 13 ka^11^. Four of these flank instabilities are defined as large lateral collapses in a study by Tibaldi^12^, with volumes ranging from 0.73 ± 0.22 km^3^ for the youngest collapse and 2.23 ± 0.97 km^3^ for the oldest. On Tenerife, at least five large sector collapses have occurred over the last 1 Ma^13,14^ with a combined deposit volume estimated at 1000 km^3^. Finally, there is strong evidence for similar flank collapse episodes on El Hierro, which is the youngest of the Canary Islands^15,16^. Multiple large sector collapses are thought to give rise to the modern-day three-armed shape of the island, the largest of which is the El Golfo collapse and occurred approximately 15000 years ago^17^ with an estimated volume of 150 km^3^ for the avalanche deposit^18^.

## Experimental method and materials tested

The materials collected were a basalt from El Hierro (EHB), a phonolite from Teide (TB) and a Paleostromboli lava^19^ from Stromboli (SB). The EHB material is a massive blocky lava containing subhedral pyroxene phenocrysts, but no vesicles visible in hand specimen (less than approximately 0.5 mm). The pyroxene phenocrysts are up to 3 mm in size (subhedral to euhedral), with some thin aligned cracks and fissures also visible. The TB unit is a light grey phonolite; there are no obvious microcracks or vesiculation, and the materials appears homogeneous in hand specimen, coarsely porphyritic, and containing phenocrysts of pyroxene and olivine. The final rock type used was a basalt of the lower Paleostromboli lava flow (Malpasso formation) on the island of Stromboli. These basalts shows significant sub-vertical and few sub-horizontal fractures on a macroscopic scale. The greyish matrix is very fine grained with some small (mm-size) greyish-white phenocrysts are visible on a macroscopic scale. Porosity was measured for all samples using the saturation and buoyancy technique following the International Society for Rock Mechanics suggested method^20^. To be sure of full saturation, even in low porosity rock, an additional step was employed whereby the sample was initially evacuated in a vacuum chamber, before water was introduced (rather than evacuation whilst submerged). The measured porosities were 6.1% for SB, 12.3% for TB, and 1.7% for EHB. The three rock types are also largely isotropic, with values for P-wave anisotropy (expressed as difference/mean in P-wave elastic velocity measured across core diameter every 22.5 degrees) for SB, TB and EHB of 2.0%, 1.8% and 2.2% respectively.

**Figure 1:**
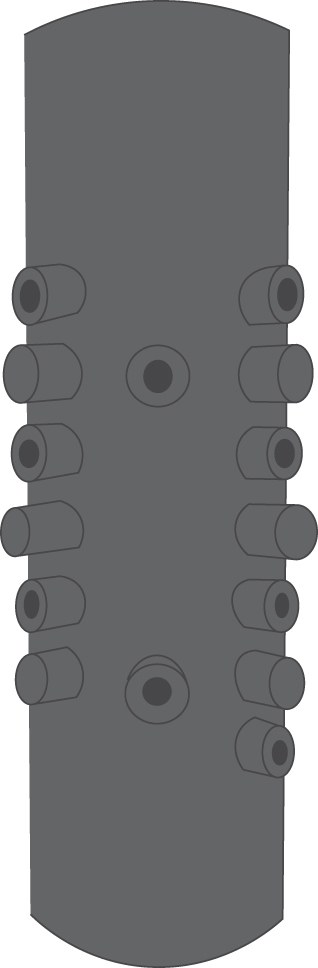
A sketch of the rubber jacket used in all experiments, showing location of AE sensors, reproduced from Fazio, 2017^25^.

**Figure 2:**
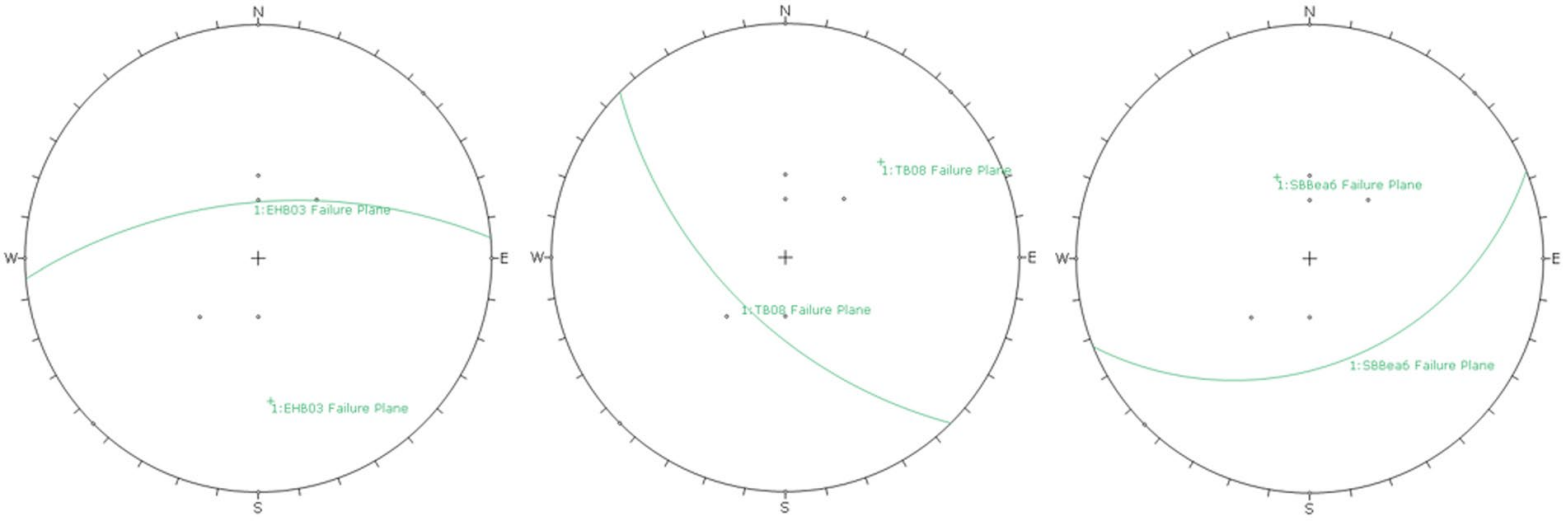
Stereographic projections of the raypaths relative to the failure plane for each of the three experiments considered.

For the rock deformation experiments, cylindrical core samples of 40 mm diameter and 100 mm length were prepared for each rock type using a diamond tipped hollow coring drill, with ends ground and lapped to a precision of 0.01 mm using a lathe combined with a cross-cut diamond wheel set up to “top off” each end of the sample. The length-diameter ratio is 2.5:1 in accordance with the ISRM suggested method^21^. Samples were dried at 100 °C for at least 3.5 hours before being vacuum saturated with water for at least 24 hours to ensure full saturation during the experiment. Experiments were performed using a Sanchez Technologies triaxial cell^22^, capable of confining pressures and pore pressures to a maximum of 100 MPa. Samples were deformed using a constant strain rate of 10^−5^ s^−1^, a confining pressure of 20.8 MPa and a pore pressure of 8.8 MPa, simulating a depth of approximately 800 m, consistent with models invoking brittle failure mechanisms^23^. Samples are encapsulated inside a Nitrile rubber (type FKM-B) jacket designed following an earlier setup at the Rock & Ice Physics Laboratory at University College London^24^. The jacket is used to isolate the sample from the confining pressure medium (silicone oil) and is fitted with 18 ports for inserting acoustic emission (AE) sensors, of which 12 were used (Fig. 1). Our sensors were developed in-house and consist of a Piezoelectric element (PZT-5A) encapsulated in an aluminum cap and waveguide^22^, and using a tungsten and polytetrafluoroethylene (PTFE) backing material to reduce internal vibrations. Voltage output was pre-amplified by 40 dB amplifiers (Itasca-IMAGE) before being digitized at 10 MHz (16 bit) and recorded to disk. A common co-ordinate frame was used throughout, ensuring that the sample (and thus the final failure plane) is orientated within the jacket (Fig. 2).

For this study, the sensors are operated in “active” mode whereby each of the 12 sensors is pulsed in sequence with a 200 V square wave, allowing the velocity of each raypath to be calculated from time of flight to the remaining 11 sensors. To improve signal to noise ratio, a stack of 16 pulses was averaged per pulsing channel. Surveys are conducted every minute allowing the damage as ‘seen’ by each raypath via the reduction in each raypath velocity, as well as inverting these data for an apparent crack density. Velocity and velocity anisotropy is highly sensitive to the original damage state of the sample and its associated porosity^26,27^. However, to derive an even more sensitive velocity ***change*** with time, in addition to the basic time of flight approach for each raypath and for each time step, a cross correlation method was employed. This works by setting a ‘master’ survey (an entire sequence of 12 pulses across the channels with the associated arrivals recorded on the non-pulsing channel) and picking an arrival time manually. Each subsequent survey then has an approximate arrival time allocated, together with a time window centered on this arrival time. A cross correlation algorithm then time shifts the survey waveforms (maximum defined by the window) so as to achieve a minimum cross-correlation to the master survey – this time is then the difference in time compared to the master event with equates to a velocity change. This method achieves extremely high accuracy of velocity change of approximately 2 m/s.

**Figure 3:**
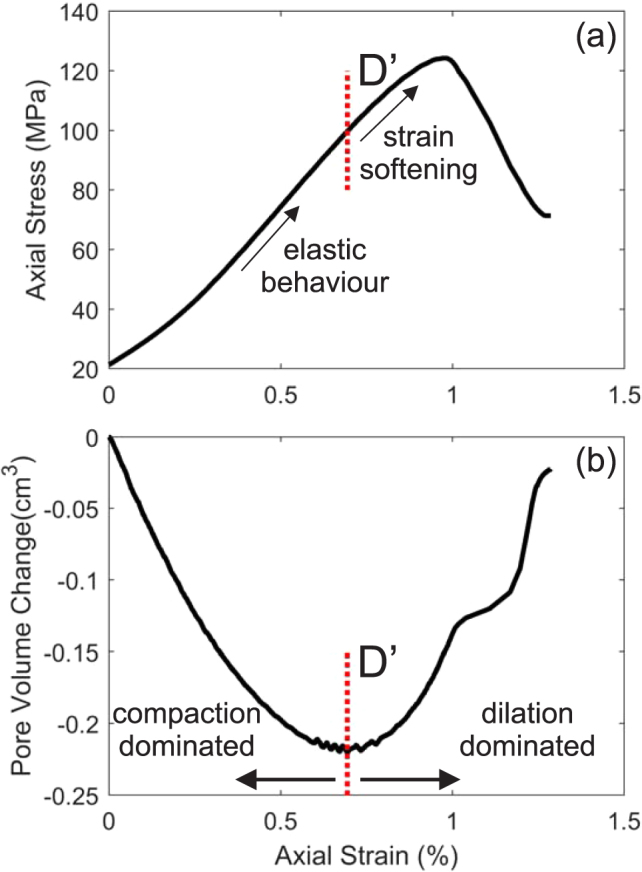
(**a**) Example stress-strain data for the TB experiment, and (**b**) the associated pore volume change. D’, the onset of strain softening, is shown by a dotted line in both.

Finally an apparent crack density, with respect to the known fracture plane orientation and rock initial porosity, is calculated using the Kuster and Toksöz effective medium theory^28,29^, which models the crack porosity as a new scattering source, injecting additional cracks as needed to match the apparent reduction in bulk modulus until the measured velocity is achieved. For our deformation experiments, we additionally normalize this by taking a minimum apparent crack density to occur at the point D’ (the onset of strain softening) of the deformation experiment. This point denotes the end of the linear elastic part of the experiment, where the additional application of a principle stress preferentially re-opens suitably orientated cracks, as well as generating new cracks (Fig. 3)^4,30,31^.

## Results

**Figure 4:**
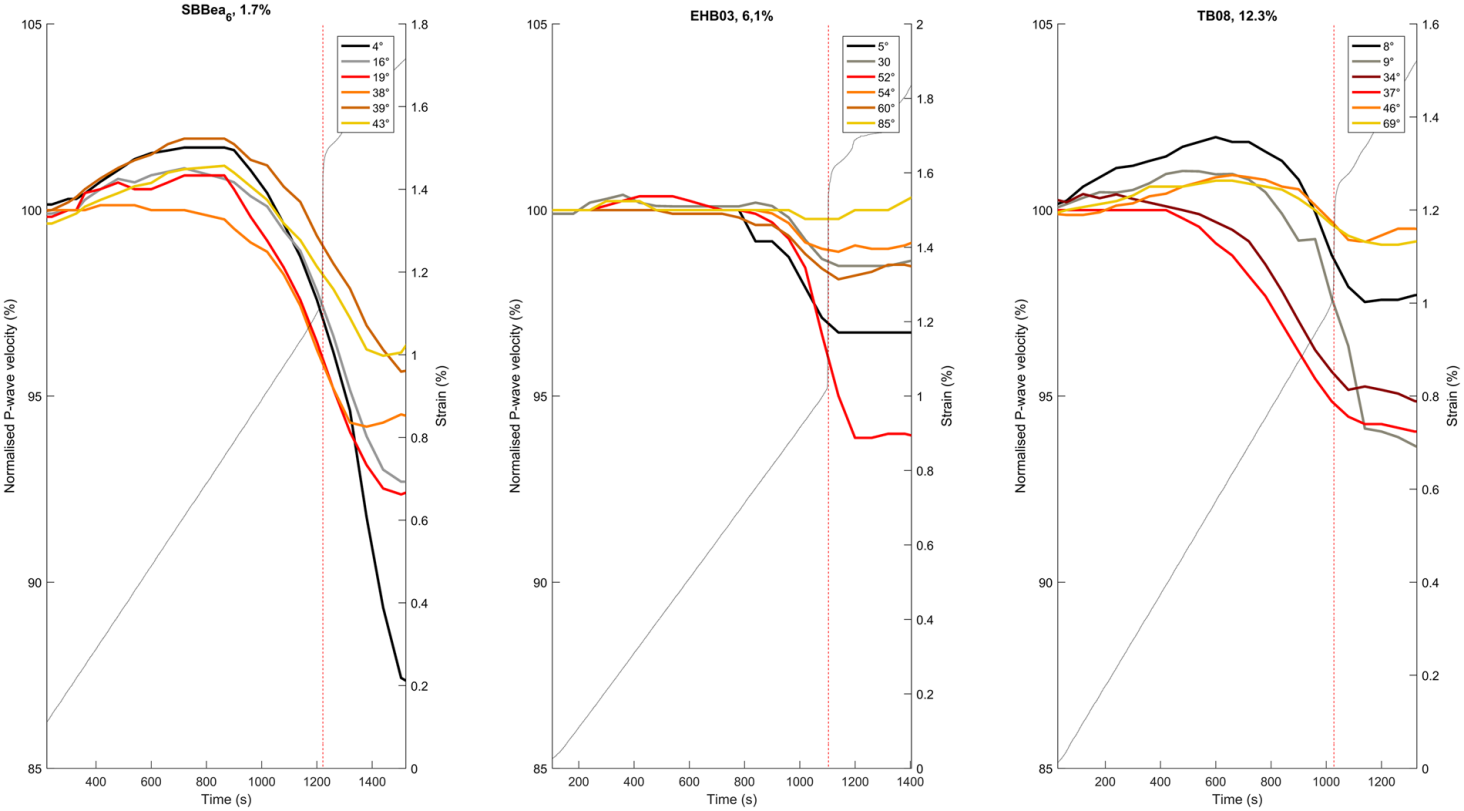
Normalised P-wave velocity as a percentage of the P-wave velocity at the start of loading, shown as a function of time from start of loading. Different raypaths indicated by the angle of intersection between the raypath and the failure plane. Grey dashed line shows strain in each experiment, whilst vertical red dotted line marks the point of sample failure (determined by a stress drop). Each plot shows 1000 seconds before failure and 300 seconds after failure.

Triaxial deformation data show strengths of 232.6 MPa, 317.5 MPa, and 124.3 MPa for SB, EHB and TB respectively. Figure 4 shows the relative velocity change along different raypaths crossing the damage zone as a function of time. For SB (porosity of 1.7%), we measure a peak in the elastic wave velocity at approximately 600 seconds prior to failure and a marked decrease in velocity is seen from approximately 200 seconds before sample failure (stress drop), which occurs at 1220 s (marked by a vertical dashed line). For the EHB data (porosity of 6.1%), there is again a noticeable decrease in elastic wave velocity measured approximately 200 seconds before failure. Finally for TB (porosity of 12.3%), a similar trend is seen, but starting (as per SB) with an increase in velocity before failure, followed by a decrease as the sample fails. The increase in velocity commences almost immediately as the sample is loaded, taking some 650 s to reach maximum (in the case of raypath at 8 degrees) followed by a velocity decrease that occurs earlier (400 seconds before failure) than in SB or EHB. Full stress-strain curves and equivalent velocity-strain plots can be found in Supplementary Fig. S1.

Regardless of the rock type we note that the largest velocity decrease (15% from the start of loading until 300 seconds after failure) is seen in the raypath that is at the shallowest angle to the fault plane (with the exception of the 5° and 30° raypaths of the EHB sample, which exhibited more scatter that other experiments), where the raypath is likely to spend the most time travelling through the damage zone. Oblique raypaths at high angles to the fault (e.g. 39°, 85° and 69° for SB, EHB, and TB respectively) experience only small reductions (<5%) in velocity during the same time, likely as a result of spending less time in the heavily damaged fracture zone. We also note that the largest time period over which a decrease in velocity is observed is found in the sample with the highest porosity.

**Figure 5:**
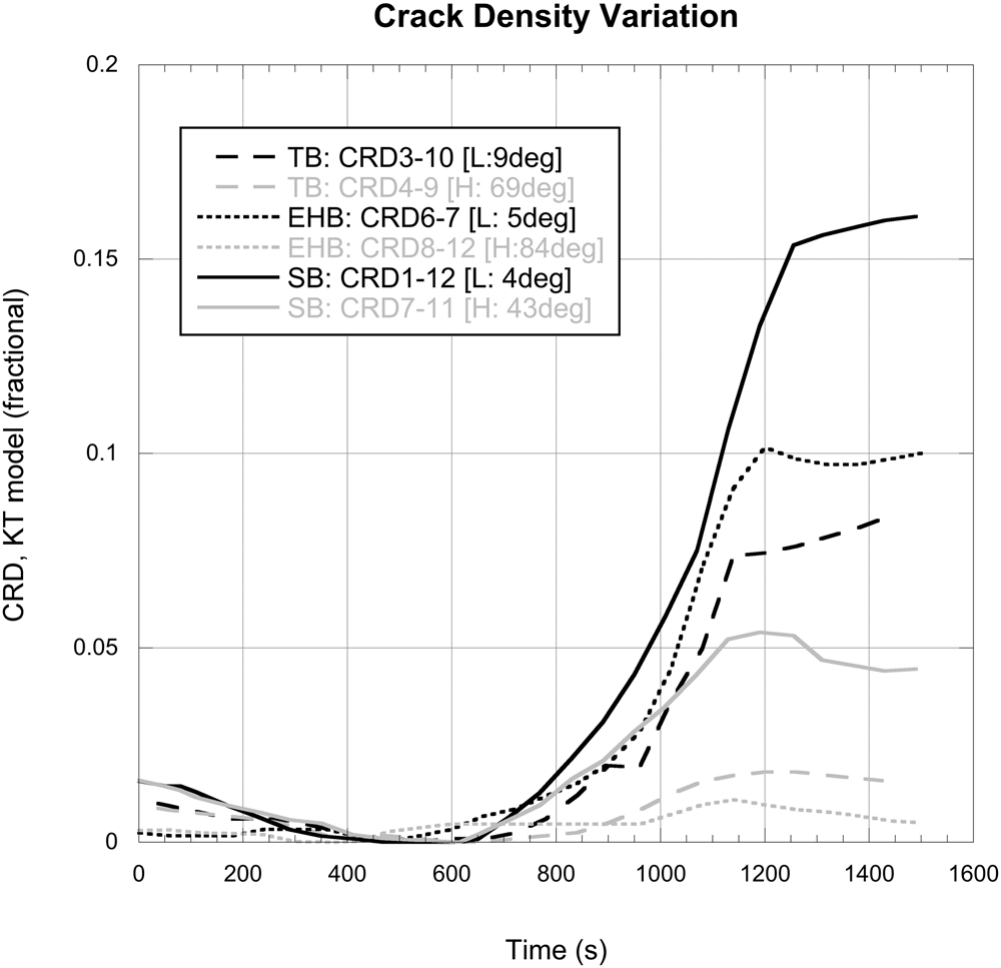
Crack density calculated by the *Kuster and Toksöz* effective media model for the minimum and maximum raypath-failure plane intersection angles, for each rock type. H = highest angle (grey) and L = lowest angle (black).

To calculate crack density from these data we applied the effective medium model of *Kuster and Toksöz*^28^, normalized to the D’ time. By normalizing to this time, the procedure above outputs a low or zero crack density at this time. Figure 5 reports the data as applied to the three rock types in this study and taking the minimum and maximum incident raypath with respect to the known faults plane in each case, shown as L (low) and H (high) in each case. In all cases the crack density starts from an initial low value, initially decreasing to a minimum due to the closure of pre-existing cracks upon application of stress, and then increasing as damage is induced by the constant strain deformation experiment. However, it is notable that each rock type behaves differently, and with a different character before and after the D’ point. Initially, EHB has the lowest crack density with low and high incident angle having similar values (0 and 0.003 respectively). The same effect is evident for TB with low and high crack densities of 0.009 and 0.011 respectively, followed by SB with low and high crack densities of 0.015 and 0.018 respectively. The data remain clustered by rock type until the D’ where upon they diverge significantly. In particular, we note two post-D’ responses. Firstly, the raypath at a low incidence in each cases is significantly more affected by the formation of the damage plane, with crack densities increasing to 0.08, 0.1 and 0.17 for TB, EHB and SB respectively; the increase in crack density for raypaths at a high incidence angle to the failure plane being far less significant. Secondly, the crack density (and therefore the nature of the damage zone) appears to be the factor controlling the velocity divergence, as the spread of the velocity divergence no longer has any relationship to the starting rock type.

## Discussion and Conclusions

Assessing the stability of natural structures such as volcanic edifices is non-trivial. In the case of active volcanoes this includes their dynamic nature, by definition, and even for dormant or extinct volcanoes the impact of weathering may have a significant effect on edifice stability. For regions that are the topic of active studies, various tools are employed, ranging from LiDAR, to InSAR, and local geophysical methods such as microseismic arrays as well as active seismic methods. These have a dual use of imaging the underlying structure as well as to derive a velocity and attenuation model. Indeed, the velocity model is a crucial first step to seismic imaging and is used extensively^6,7,9^. In this study, we have sought to test the sensitivity of these seismic imaging tools by conducting a laboratory study to explore the variation of P-wave velocity with raypath orientation with regard to a newly developing fracture damage zone.

Such a scenario has particular application in volcanically active regions, ranging from edifice stability to calderas. The three rock types used here are all associated with volcanoes exhibiting a history of flank collapses (El Hierro, Stromboli, and Tenerife) and span a range of ages in regards to their last major episode of instability. During the triaxial deformation, the P-wave velocity decreases markedly as cracks nucleate and form a pervasive damage zone. Velocities are particularly affected when they travel at a shallow angle through the damage zone. Previous studies have often used Acoustic Emission data to map this development in 4D^10^. Here we take the active P-wave surveys to achieve a similar aim, but with the advantage of relating the data to physical parameters, in this case the apparent crack density that the raypath interacts with as it propagates through the sample. For instance, lower porosity (high incidence) data appear less affected by the damage zone generation (although due to the uncontrolled nature of the damage zone orientation with respect to the sensor array, this is partly attributed to the value of raypath incidence). This effect if further modified by the initial porosity where, for example, TB data (12.3% porosity), shows a velocity decrease as early as 1000 seconds before failure, suggesting that the onset of damage zone evolution and preferred failure orientation in the sample can occur just 200 seconds after the application of the principal stress.

**Figure 6:**
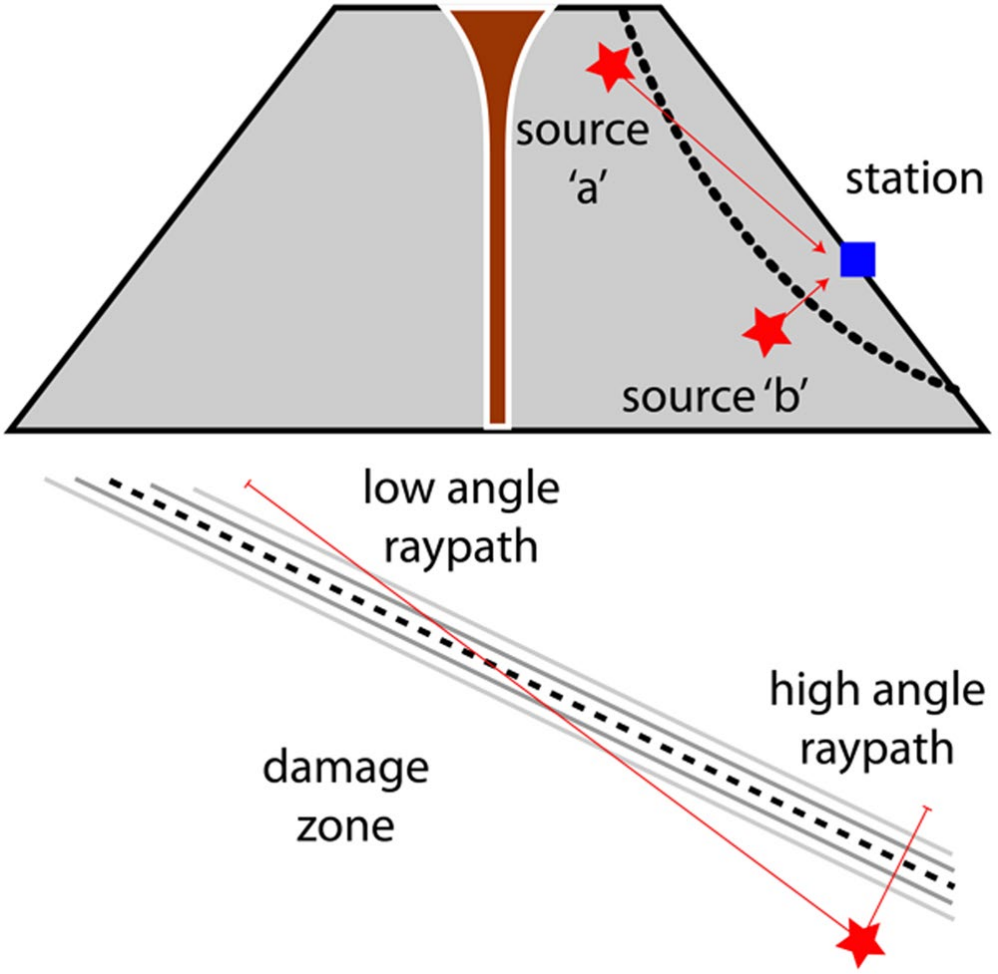
(Top) Conceptual model illustrating an idealized raypath (red) through a damage zone (dotted lines) in an unstable edifice. (Bottom) illustration highlighting raypath transit length through the damage zone as a result of incidence angle.

Conversely, the initial porosity appears to have very little effect on the final spread of the velocity data at the ***end*** of each experiment. For the lowest porosity SB (1.7%), the normalized velocity ranges from 85% to 97%, for EHB (6.1%) ranging from 94% to 100% and for TB (12.3%) spanning the range 93% to 99%. However, some subtle differences are evident: the SB and TB rock types have a more prominent porosity texture (vesiculation) compared to EHB and this likely gives rise to the larger increase in velocity seen in Fig. 4 – in both cases increasing from around 100% to 102%. This increase in velocity is caused by the closure of pre-existing intergranular cracks by the maximum principal stress^4^. The data across all the rock types show that low incidence raypaths are more greatly affected than those at high angles, as previously noted. However, there is some variation to this general trend, which is likely the result of the natural heterogeneity each individual raypath intersects with once the fracture has been generated (Fig. 6).

Calculating crack density offers a way of relating the elastic wave velocity data (P-wave) to a physical mechanism – in this case by injecting a population of ‘penny’ shaped cracks sufficient to reduce the velocity by the measured amount. The *Kuster and Toksöz* model is an effective medium model that considers the scattering effect of a passing wave-field though a solid with embedded cracks. By relating the degree of scattering to the density of cracks, an apparent reduction in velocity is derived. The method assumes a linear elastic material (i.e. the model does not allowing pore/crack collapse) and is limited to a relatively dilute concentration of pores, so that the porosity cannot be too high. For the rocks used here, this is a reasonable limitation. Using this analysis, the crack density starts with the three rock types clustered by type (Fig. 5), but during deformation and the creation of the failure pane, ends with each trend clustering by raypath incidence angle. Although only the end members (lowest and highest incident angle) are taken, and given that we deal with a ‘natural’ fault plane resulting in some scatter between raypaths (which cannot be accurately set with respect to their orientation to that eventual failure plane), this switch nonetheless provides evidence that the initial porosity is largely homogeneous and if it contains a crack network, then these cracks are likely to be randomly orientated. After the fault is formed, a preferred orientation of damage is generated, which appears in the elastic wave velocity response as a significant anisotropy both in crack density and in raw velocity change. Such an approach would usefully be combined with regional tectonic or structural data to reveal any pre-existing faulting or damage planes, as is clear in our experiments. In this dataset, a particularly good example is the raypath EHB 6–7 which increases from the lowest CRD to the second highest purely through the generation of a fresh damage zone that is also highly aligned to the raypath. However, it should also be noted that the zero value reported for crack density (which occurs at a time of approximately 500 s) is due to normalization: it is possible for a physical non-zero value, but taking a normalized approach allows the variation to be seen clearly without calibration via crack counting.

We conclude that: (1) P-wave elastic velocities are a good proxy for the evolution of damage, as tested in basalt from El Hierro and Stromboli, and a phonolite from Tenerife, and that these three rock types are largely isotropic until the initiation of the development of the damage zone which occurs after D’ has been reached; (2) the measured velocity along a given raypath is highly sensitive to the angle of incidence to any fracture damage zone encountered; and (3) the apparent crack density (as seen by the individual raypaths) produces a physical representation of the degree of fracturing required to reduce the velocity by the required proportion, and that velocity and velocity anisotropy are disproportionally affected by the generation of the fresh damage zone. Developing this further, we conclude that the regional stress regime (simulated by sigma 1 in our experiments) generates a significant anisotropy that the rocks did not previously possess (Fig. 3). Such laboratory data, backed up with known planes of weakness in the edifice of many volcanoes, suggests that active seismic surveys in field settings need to be processed and interpreted with particular care. This is likely to be especially important for surveys that propagate through any aligned crack networks of fault zones, as suggested, for example, in the regional tectonic stresses of Stromboli volcano aligned along a NE-SW weakness zone in that case^32^. However, such data, if properly referenced to the intact rock data and calibrated via rock physics changes with depth, might also elucidate useful data relating to crack density and anisotropy variation at depth, providing new knowledge of the regional tectonic environment.

### Data Availability

All laboratory data generated by the experiments conducted in this study are available from the corresponding author by request.

## Electronic supplementary material

Supplementary Figure S1
