# Supplementary material for: Fracture and damage localization in volcanic edifice rocks from El Hierro, Stromboli and Tenerife
Source: Sci Rep. 2018 Jan 31;8:1942. doi: 10.1038/s41598-018-20442-w (PMC5792434; doi:10.1038/s41598-018-20442-w)
Supplement: Supplementary file 1 — Supplementary Figure S1 [file 41598_2018_20442_MOESM1_ESM.pdf]

Fracture and damage localization in volcanic edifice rocks from El Hierro, Stromboli and Tenerife

Claire E. Harnett*[1], Philip M Benson[2], Pete Rowley[3] and Marco Fazio[2]

Supplementary Material Figure S1

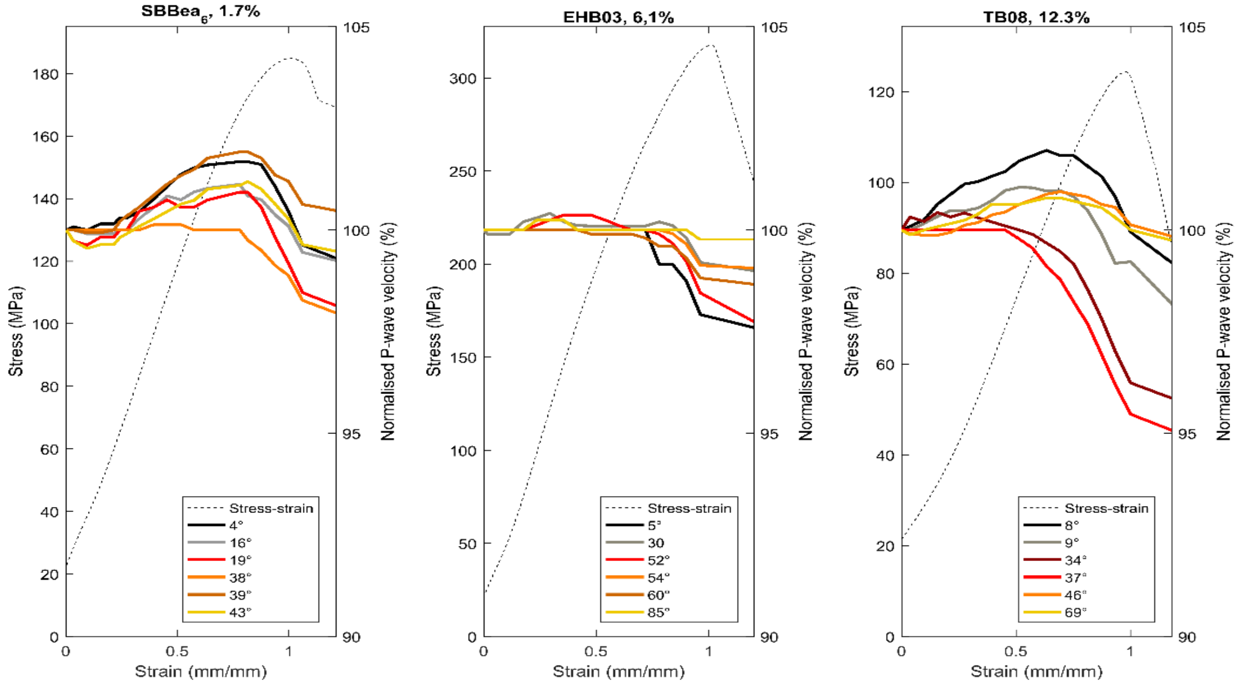

Normalised P-wave velocity as a percentage of the P-wave velocity at the start of loading, shown as a function of strain. Different raypaths indicated by the angle of intersection between the raypath and the failure plane. Dashed line shows mechanical stress-strain in each experiment, and the plots are clipped to 0.2mm/mm after peak stress.
